# Supplementary material for: Data on Connection With the Natural Environment and Its Impact on Mental Health Among Allotment and Non-Allotment Owners
Source: J Open Psychol Data. 2024 Sep 13;12:9. doi: 10.5334/jopd.122 (PMC12270269; doi:10.5334/jopd.122)
Supplement: Appendix 1. — Online survey used. [file jopd-12-122-s1.pdf]

Appendix 1 - Online survey used

Instructions

Thank you for taking the time to complete this survey, please answer the questions as honestly as you can, and read each set of instructions carefully.

Section 1: About you and your health.
Age: _______
Gender: ____________________
Nationality: ______________________
How many hours on average do you currently spend in nature per week? For example, this could be but isn't limited to visiting parks, walks, gardening, and allotment work:
____

What activities in nature do you participate in? (For example, walks or gardening, please list as many as apply to you:
____
____
____
____

Do you currently own/ work on an allotment site? _______________
Do you currently participate in an allotment group/project? _________

Below are some statements about feelings and thoughts.
Please tick the box that best describes your experience of each over the last 2 weeks

	None of the time	Rarely	Some of the time	Often	All of the time
I've been feeling optimistic about the future					
I've been feeling useful					
I've been feeling relaxed					
I've been dealing with problems well					
I've been thinking clearly					

I’ve been feeling close to other people					
I’ve been able to make up my own mind about things					

Please tick the box you feel most applies to you.

	1 Not very true of me	2	3	4	5 Very true of me
I have high self-esteem					

These questions relate to your physical health. Please tick the box you think best represents how you feel about your physical health. Physical health refers to your general state of health and wellbeing and your ability to perform daily activities.

	Excellent	Very good	Good	Fair	Poor
In general, how would you say your health is….?					
Compared to others your age, would you say your health is...?					

Section 2: Your thoughts about nature

For each of the following, please rate the extent to which you agree with each statement, using the scale from 1 to 5 as shown below. Please respond as you really feel, rather than how you think "most people" feel.

	1	2	3	4	5
	Disagree strongly	Disagree a little	Neither agree or disagree	Agree a little	Agree strongly
My ideal vacation spot would be a remote, wilderness area					
I always think about how my actions affect the environment.					
My connection to nature and the environment is a part of my spirituality.					
I take notice of wildlife wherever I am.					
My relationship to nature is an important part of who I am.					
I feel very connected to all living things and the earth.					

Section 3: About the people around you

Please specify how much you disagree or agree with each statement concerning you and your group. Please tick one box in each line below. Your group could be any group/s you are a part of (for example: allotment group, student group, work group)

	I strongly disagree,	I disagree,	I slightly disagree	I neither agree nor disagree	I slightly agree	I agree	I strongly agree
I feel a bond within my (group)							
I feel similar to the other members of my (group)							

I have a sense of belonging to my (group)							
I have a lot in common with the members of my (group)							

Please read the statements and tick the box you feel best describes you. In answering the questions it is best to think of your life as it generally is now.

	Yes	More or less	No
I experience a general sense of emptiness			
There are plenty of people I can rely on when I have problems			
There are many people I can trust completely			
I miss having people around			
There are enough people I feel close to			
I often feel rejected			

Please tick one box in each row that best describes how you feel.

	1<br>Not at all	2	3	4	5	6	7<br>Completely
Do you get the emotional support you need from other people?							

Do you get the help you need from other people?							
Do you get the resources you need from other people?							
Do you get the advice you need from other people?							

Please tick the box which best describes you.

	Not true at all<br>1	Hardly true<br>2	Moderately true<br>3	Exactly true<br>4
I can remain calm when facing difficulties because I can rely on my coping abilities				
I am confident that I could deal efficiently with unexpected events				
I can always manage to solve difficult problems if I try hard enough				
If I am in trouble, I can usually think of a solution				
If someone opposes me, I can find the means and ways to get what I want				
