# Supplementary material for: Data on Connection With the Natural Environment and Its Impact on Mental Health Among Allotment and Non-Allotment Owners
Source: J Open Psychol Data. 2024 Sep 13;12:9. doi: 10.5334/jopd.122 (PMC12270269; doi:10.5334/jopd.122)
Supplement: Supplementary File. — Coding sheet for dataset. [file jopd-12-122-s2.pdf]

Section/Question Number	Section title	Code	Item	values
S1 Q1	demographics	D_Age	Age	Open ended
S1 Q1	demographics	D_Gender	Gender	Open ended<br>(coded as follows:)<br>1=man<br>2=woman
S1 Q1	demographics	D_Nation	Nationality	Open ended
S1 Q1	demographics	D_hours	How many hours on average do you currently spend in nature per week? For example, this could be but isn't limited to visiting parks, walks, gardening, and allotment work:	Open ended
S1 Q1	demographics	D_activities	What activities in nature do you participate in? (For example, walks or gardening, please list as many as apply to you:	Open ended
S1 Q1	demographics	D_allot	Do you currently own/ work on an allotment site?	Yes=1<br>No=2
S1 Q1	demographics	D_group	Do you currently participate in an allotment group/project?	Yes=1<br>No=2

S1 Q2	Mental Health	MH_1	I've been feeling optimistic about the future	1=none of the time<br>2=rarely<br>3=some of the time<br>4=often<br>5=all of the time
S1 Q2	Mental Health	MH_2	I've been feeling useful	1=none of the time<br>2=rarely<br>3=some of the time<br>4=often<br>5=all of the time
S1 Q2	Mental Health	MH_3	I've been feeling relaxed	1=none of the time<br>2=rarely<br>3=some of the time<br>4=often<br>5=all of the time
S1 Q2	Mental Health	MH_4	I've been dealing with problems well	1=none of the time<br>2=rarely<br>3=some of the time<br>4=often<br>5=all of the time
S1 Q2	Mental Health	MH_5	I've been thinking clearly	1=none of the time<br>2=rarely<br>3=some of the time<br>4=often<br>5=all of the time
S1 Q2	Mental Health	MH_6	I've been feeling close to other people	1=none of the time<br>2=rarely

				3=some of the time<br>4=often<br>5=all of the time
S1 Q2	Mental Health	MH_7	I've been able to make up my own mind about things	1=none of the time<br>2=rarely<br>3=some of the time<br>4=often<br>5=all of the time

S1 Q3	Self-Esteem	SE_1	I have high self-esteem	1= not very true of me<br>2<br>3<br>4<br>5=very true of me

S1 Q4	Physical health	PH_1	In general, how would you say your health is….?	1=excellent<br>2=very good<br>3=good<br>4=fair<br>5=poor
S1 Q4	Physical health	PH_2	Compared to others your age, would you say your health is...?	1=excellent<br>2=very good<br>3=good<br>4=fair<br>5=poor

S2 Q5	Connectedness to nature	Nat_1	My ideal vacation spot would be a remote, wilderness area	1= disagree strongly<br>2=disagree a little<br>3=neither agree or disagree<br>4=agree a little<br>5=agree strongly
S2 Q5	Connectedness to nature	Nat_2	I always think about how my actions affect the environment.	1= disagree strongly<br>2=disagree a little<br>3=neither agree or disagree<br>4=agree a little<br><br>5=agree strongly
S2 Q5	Connectedness to nature	Nat_3	My connection to nature and the environment is a part of my spirituality.	1= disagree strongly<br>2=disagree a little<br>3=neither agree or disagree<br>4=agree a little<br><br>5=agree strongly
S2 Q5	Connectedness to nature	Nat_4	I take notice of wildlife wherever I am.	1= disagree strongly<br>2=disagree a little<br>3=neither agree or disagree<br>4=agree a little

				5=agree strongly
S2 Q5	Connectedness to nature	Nat_5	My relationship to nature is an important part of who I am.	1= disagree strongly<br>2=disagree a little<br>3=neither agree or disagree<br>4=agree a little<br><br>5=agree strongly
S2 Q5	Connectedness to nature	Nat_6	I feel very connected to all living things and the earth.	1= disagree strongly<br>2=disagree a little<br>3=neither agree or disagree<br>4=agree a little<br>5=agree strongly

S3 Q6	Social Identity	SocID_1	I feel a bond within my (group)	1=I strongly disagree<br>2=I disagree<br>3=I slightly disagree<br>4=I neither agree nor disagree<br>5=I slightly agree<br>6=I agree<br>7= I strongly agree
S3 Q6	Social Identity	SocID_2	I feel similar to the other members of my (group)	1=I strongly disagree<br>2=I disagree<br>3=I slightly disagree<br>4=I neither agree nor disagree<br>5=I slightly agree<br>6=I agree<br>7= I strongly agree
S3 Q6	Social Identity	SocID_3	I have a sense of belonging to my (group)	1=I strongly disagree<br>2=I disagree<br>3=I slightly disagree<br>4=I neither agree nor disagree<br>5=I slightly agree<br>6=I agree<br>7= I strongly agree
S3 Q6	Social Identity	SocID_4	I have a lot in common with the members of my (group)	1=I strongly disagree<br>2=I disagree<br>3=I slightly disagree<br>4=I neither agree nor disagree<br>5=I slightly agree<br>6=I agree<br>7= I strongly agree

S3 Q7	Loneliness	Lon_1	I experience a general sense of	1=yes

			emptiness	2=more or less<br>3=no
S3 Q7	Loneliness	Lon_2	There are plenty of people I can rely on when I have problems*	1=yes<br>2=more or less<br>3=no
S3 Q7	Loneliness	Lon_3	There are many people I can trust completely*	1=yes<br>2=more or less<br>3=no
S3 Q7	Loneliness	Lon_4	I miss having people around	1=yes<br>2=more or less<br>3=no
S3 Q7	Loneliness	Lon_5	There are enough people I feel close to*	1=yes<br>2=more or less<br>3=no
S3 Q7	Loneliness	Lon_6	I often feel rejected	1=yes<br>2=more or less<br>3=no

***= reverse coded**

S3 Q8	Social Support	SocSup_1	Do you get the emotional support you need from other people?	1= not at all<br>2<br>3<br>4<br>5<br>6<br>7= completely
S3 Q8	Social Support	SocSup_2	Do you get the help you need from other people?	1= not at all<br>2<br>3<br>4<br>5<br>6<br>7= completely
S3 Q8	Social Support	SocSup_3	Do you get the resources you need from other people?	1= not at all<br>2<br>3<br>4<br>5<br>6<br>7= completely
S3 Q8	Social Support	SocSup_4	Do you get the advice you need from other people?	1= not at all<br>2<br>3<br>4<br>5<br>6<br>7= completely

S3 Q9	Self-Efficacy	SEff_1	I can remain calm when facing difficulties because I can rely on my coping abilities	1= not true at all<br>2=hardly true<br>3=moderately true<br>4= exactly true
S3 Q9	Self-Efficacy	SEff_2	I am confident that I could deal efficiently with unexpected events	1= not true at all<br>2=hardly true<br>3=moderately true<br>4= exactly true
S3 Q9	Self-Efficacy	SEff_3	I can always manage to solve difficult problems f I try hard enough	1= not true at all<br>2=hardly true<br>3=moderately true<br>4= exactly true
S3 Q9	Self-Efficacy	SEff_4	If I am in trouble, I can usually think of a solution	1= not true at all<br>2=hardly true<br>3=moderately true<br>4= exactly true
S3 Q9	Self-Efficacy	SEff_5	If someone opposes me, I can find the means and ways to get what I want	1= not true at all<br>2=hardly true<br>3=moderately true<br>4= exactly true
